# Versatile Multimodality Imaging System Based on Detectorless and Scanless Optical Feedback Interferometry—A Retrospective Overview for A Prospective Vision

**DOI:** 10.3390/s20205930

**Published:** 2020-10-20

**Authors:** Massimo Brambilla, Lorenzo Luigi Columbo, Maurizio Dabbicco, Francesco De Lucia, Francesco Paolo Mezzapesa, Gaetano Scamarcio

**Affiliations:** Dipartimento di Fisica “Michelangelo Merlin”, Università degli Studi e Politecnico di Bari, and IFN CNR, sede di Bari, via Amendola 173, 70126 Bari, Italy; Massimo.Brambilla@poliba.it (M.B.); lorenzo.columbo@polito.it (L.L.C.); fdl1c13@soton.ac.uk (F.D.L.); francesco.mezzapesa@stiima.cnr.it (F.P.M.); gaetano.scamarcio@uniba.it (G.S.)

**Keywords:** optical feedback interferometry, laser self-mixing, semiconductor lasers

## Abstract

In this retrospective compendium, we attempt to draw a “fil rouge” along fifteen years of our research in the field of optical feedback interferometry aimed at guiding the readers to the verge of new developments in the field. The general reader will be moved at appreciating the versatility and the still largely uncovered potential of the optical feedback interferometry, for both sensing and imaging applications. By discovering the broad range of available wavelengths (0.4–120 μm), the different types of suitable semiconductor lasers (Fabry–Perot, distributed feedback, vertical-cavity, quantum-cascade), and a number of unconventional tenders in multi-axis displacement, ablation front progression, self-referenced measurements, multispectral, structured light feedback imaging and compressive sensing, the specialist also could find inspirational suggestions to expand his field of research.

## 1. Introduction

A number of excellent reviews [1,2,3,4,5], two books [6,7], and a few book chapters [8,9,10], among the others, have been published on the subject of laser self-mixing, as it was initially named, or optical feedback (OF) interferometry, as it is more often termed today.

OF happens when part of the radiation emitted by a laser source is coupled back to the laser cavity before losing coherence. It happens, or may happen, in all experiments with a laser source if no specific care is taken in order to avoid it. OF always pushes the laser emission to change its instantaneous values of frequency and power. Whether it is a trouble or a benefit, mostly depends on the way we look at. It is a trouble for the stability of laser emission if the OF is uncontrolled; it can be a benefit when OF is under the researcher’s control and intended for some purpose; for example, linewidth narrowing [11], chaos synchronization [12] or sensing applications [13]. In this revisitation of our research we concentrate on the latter benefit: the changes induced by OF on the instantaneous laser frequency and power bring information on the optical properties of the whole system, the laser itself, the target responsible of the back scattering (or back reflection), and the space in between. The whole system is a nonlinear optoelectronic system, possibly experiencing regimes of dynamical instability interspaced with regimes of stationary behavior. Most of the practical sensing schemes based on OF analyze the information conveyed by the variations of the stationary parameters caused by modifications of the radiative field amplitude and phase occurred along the optical path forth and back from the target.

Information density conveyed by field spatial frequency (*k*) increases in going from a collimated to a focused beam and can be tailored by structuring the optical field by means of spatial light modulators. Spatial frequency relates to image resolution and OF imaging has demonstrated the potential to beating the diffraction limit [14,15]. On the other hand, the natural reference unit of interferometric measurement is the fringe, so that fringe-counting is the simplest possible signal analysis. However, it is limited to quantized results multiple of the laser half-wavelength *λ*/2 or the inverse of the laser linewidth Δ*ν* (Δ*ϕ* = 2π = 2π/*λ*2Δ*x* = 2πΔ*νt* −> Δ*x* = *λ*/2 or *t* = 1/Δ*ν*). Better sensitivity can be achieved by phase retrieval algorithms [16] or phase-locking techniques [17], and OF is challenged by reaching 10^−4^*λ* resolution [18].

**Figure 1:**
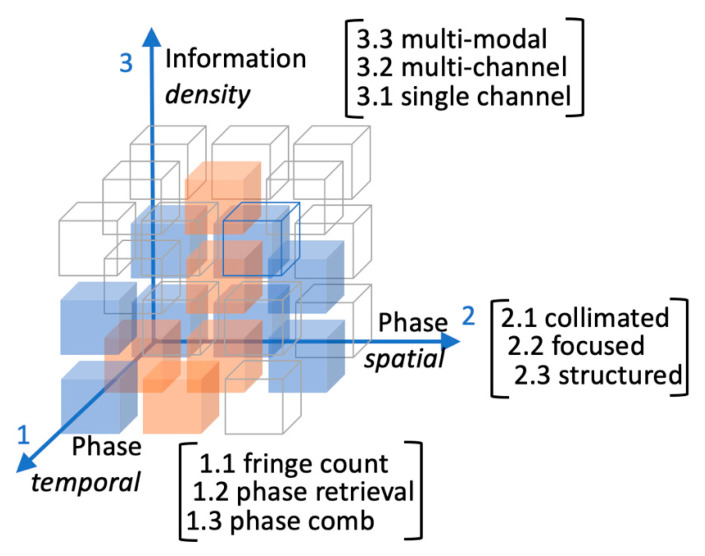
A suggestive drawing of the optical feedback (OF) development space. Axes 1 and 2 measure the information increase due to improving temporal and spatial resolution. Along axis 3 the information content achievable with more sophisticated OF setups. Blue cubes make the content of this article. Orange cubes are regions explored by other groups cited in the text.

The Rubik-like cube illustrated in Figure 1 symbolizes our idea of the OF expansion capability. We represented the versatility of OF systems on the third axis: from the simplest single-channel to multiple parallel channels, OF has matured the chance of going multi-modal [19,20,21]. Axes 1 and 2 measure the information increase due to improving time and space resolution.

We have no conceit to add any better word on the topic we have been engaged with for almost fifteen years. This is not a review paper then, not an orthodox one, at least. However, our research has traveled a certain number of secondary roads, making us discover new application fields and exploring theoretical aspects that have remained in the shadows.

This review is mostly intended for the general reader that could find valuable ideas off the mainstream research in OF interferometry, but we believe that also the experts in the field would enjoy the unusual narrative style of the article, and possibly remind of some “forgotten secret wish” that would be the time to dust off. This review will move from Section 2 introducing the reader the essential model describing the system laser-path-target. Almost no equations are transcribed to keep the reading on a narrative pace. Section 3 begins at block (1.1, 2.1, 3.1) and we proceed sparsely to block (1.2, 2.3, 3.2), introduced in Section 4. In Section 5 we lay the groundwork on how to reach block (1.3, 2.3, 3.3) (multi-modal system with structured light illumination and self-referenced phase). The closing Section presents our ideas on the future-istic OF sensing and imaging.

## 2. Basic Principles and Modeling Equations

The laser near threshold is a highly nonlinear system quite sensitive to any perturbation of the energy density equilibrium inside the cavity between carriers and the electromagnetic field. Any radiation coherent with the intracavity field entering the laser would disturb the dynamic equilibrium forcing the system to find a different working point. What is actually measured in OF experiments is the attempt of carriers and photons to restore the energy density balance given the feedback physical dimensions: amplitude, phase, and frequency. 

The most famous rate equation model including the effect of OF in a semiconductor diode laser (SDL) was proposed by Lang and Kobayashi in 1980 [22] and, despite its limitations, is still widely used today, also for other types of lasers. With respect to the free running laser system, the model is characterized by the feedback delay *τ* and the feedback strength *κ*. A systematic experimental verification of the Lang–Kobayashi model was made a few years later by Tkach and Chraplyvy [23] who identified five dynamical regimes in the *τ−κ* space. 

In 1984, Acket et al. [24] introduced another parameter to characterize the OF strength C = *κτε*(1+*α*^2^)^1/2^, since then renowned as the feedback parameter C, the most immediate, and quantitative, identifier of the OF dynamical regimes. C enters into play in the definition of the stationary laser emission mode frequency, with (*ω*_F_) and without (*ω*_S_) OF:

*ω*_F_*τ* = *ω*_S_*τ* − C *sin*(ω_F_*τ* + *atanα*).

Once recognized that *ωτ* = *ϕ*, this equation holds the name of excess-phase equation (EPE) and is interpreted as the phase response of the system *ϕ*_F_ to the phase stimulus *ϕ*_S_ (the phase accumulated by the emitted field in a complete round trip forth and back to the target when C = 0). The importance of EPE cannot be overestimated since its shape defines the envelope of the OF signal in almost all practical cases. In fact, both the modulation of the laser power and of the laser terminals voltage drop are demonstrated to be proportional to *cos*(*ϕ*_F_).

It is quite obvious from its own definition, that the value of C is affected by the linewidth enhancement factor (LEF) *α*, or Henry factor [25], a phenomenological parameter coupling the refractive index dependence to the gain dependence on the carrier density in semiconductor lasers. Since *α* ~ 0 in quantum-cascade lasers (QCLs) and *α* >> 0 in SDLs, the latter will have a larger C-value, the other parameters being equal, thus making SDLs typically more unstable against OF than the QCLs. In Section 3 we present evidence of exploiting the different sensitivity to OF in SDLs and in QCLs for increasing measurement resolution. The longer the temporal delay *τ* of the feedback field, the larger its effect on the phase shift is. *τ* is usually expressed in terms of the optical distance laser-target *τ* = 2*Ln⁄c*, thus designating two immediate “measurement channels”: refractive index and external cavity length.

It might be less obvious, but the target surface affects the value of C in two different ways: by its complex reflectivity, included in the definition of *κ*; and by the so-called mode-matching factor *ε*, the ratio of feedback power actually coupled back into the laser cavity because of geometrical constrains. Target related geometrical constrains, such as surface scattering and surface anisotropy should be considered as well as cavity related geometrical factors, like spatial light modulators, beam divergence or optical apertures. In the following, there will be more than one occasion to highlight the relevance of this often-overlooked parameter. 

For our purpose, we distinguish only three operative regimes [3]: (i) weak feedback (C < 1) where the amplitude of the OF fringes is proportional to the feedback power; (ii) moderate feedback (1 < C < 4.6) where the OF fringes become sawtooth-like and the system is only bi-stable; (iii) strong feedback (C >> 10) where the system is largely multi-stable, the fringes flatten, and the OF signal loses phase information. Outside of these regimes, the OF signal exhibits irregular and even chaotic fluctuations suitable to extract statistical information on the system dynamics, but unmanageable for sensing applications.

EPE is the workhorse equation for OF simulations in a large variety of experiments where the change of *ϕ*_S_(t) is slow on the time scale of carriers and photons relaxation and the optical field can be considered single mode. 

**Figure 2:**
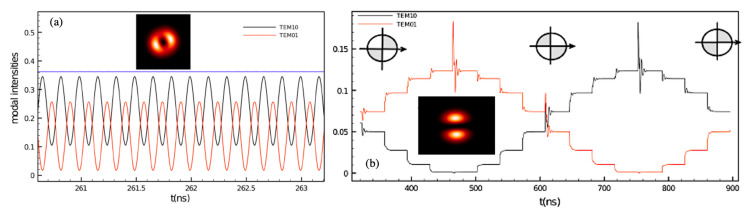
(**a**) Left: Temporal evolution of the modal intensities of the TEM10 and TEM01 modes of free-running VCSEL. The blue trace represents the sum of the modal intensities. (**b**) Time plot of the modal intensities during a feedback mask rotation from Θ = 0 (left inset mask) to Θ = π (right inset mask). The insets show image plots of the numerical simulation of the field intensity variation (adapted from Ref. [26]).

In order to address multi-mode laser dynamics, either longitudinal, transverse or polarization, we developed a more sophisticated numerical approach dealing with an extension of the Lang–Kobayashi model [26,27]. As an example of the great deal of information on the temporal and spatial evolution of the field dynamics, that can be gained from full integration of the system of coupled delayed differential equations, the simulation of a two-transverse mode Vertical Cavity Surface Emitting Laser (VCSEL) including spatially structured OF is reported in Figure 2. The model has been also extensively investigated to predict the intrinsic stability [28] and the nonlinear frequency mixing [29] in QCLs.

## 3. Single Mode Feedback Sensors

The first of the experimental sections aims at demonstrating the robustness and versatility of the OF interferometry both in SDLs and QCLs, through discussion of off-the-main-road applications like ablation monitoring and tracking of the tool-center-point in coordinate-measuring machines (CMM). We also introduce the concept of self-referenced phase (then moving two steps along axis 1 in Figure 1), that is a convenient well-regulated modulation superposed to the OF signal in order to improve the sensitivity. At the end of the section we make a side-step (along axis 2 in Figure 1) in going from collimated, or even diverging, beams to focused beams required for imaging.

### 3.1. Optical Feedback Sensing in Semiconductor Diode Lasers

As the first step along the chosen path, we show how robust is the optical feedback signal against incoherent scattering from any other light source, but the laser/detector itself. Among the several other evidences that can be found in the vast literature, one of the strongest is the signal-to-noise ratio (SNR) preserved when the target is illuminated by another laser source [30]. 

**Figure 3:**
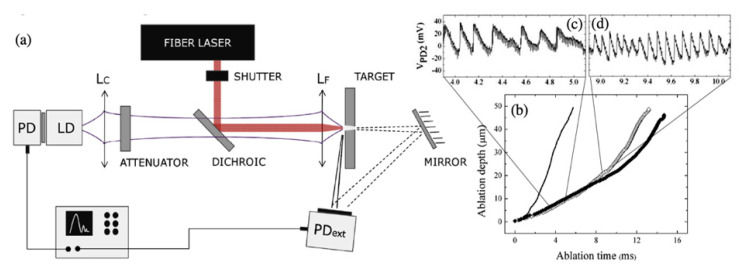
(**a**) Experimental setup of the OF ablation sensor. (**b**) Reconstructed ablation front progression from analysis of OF signal. (**c**,**d**) Representative OF fringes at different ablation rates (adapted from Reference [31]).

The OF sensor designed to monitor the ablation depth of holes drilled in stainless steel foils by a 120 ps laser pulse train is sketched in Figure 3. The OF signal preserves a good SNR of the interference fringes marking the ablation front progression in steps of *λ*_OF_/2 = 0.41 μm even when the sensing beams is aligned co-linear to the much stronger working beam from the fiber laser. Back scattered light at *λ*_W_ = 1.06 μm is residually transmitted by the dichroic beam-splitter and leaves “clock” signatures each 9 μs superposed to the OF fringes, corresponding to the 110 kHz pulse train of the ps laser, thus allowing for a much better resolution of the ablation front progression, compared to the direct fringe count.

**Figure 4:**
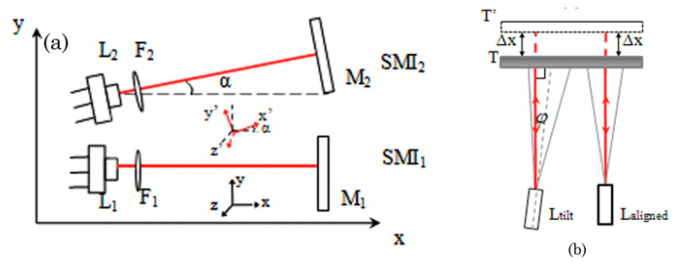
(**a**) In-plane (xy) projection of a 3D arrangement of three OF displacement sensors for simultaneous measurement of three degrees of freedom of target motion. (**b**) Grey lines draw the optic rays of a divergent laser beam, required to compensate the target tilt around the *z*-axis (adapted from Reference [32]).

The robustness of OF to cross-talking from incoherent photons allows for designing compact multiple lasers sensors capable of simultaneously performing different measurements on the same target. As an example, we reproduce in Figure 4 the arrangement of two OF displacement sensors misaligned on purpose to measure yaw and pitch rotations together with the longitudinal displacement of a sliding target [32]. 

**Figure 5:**
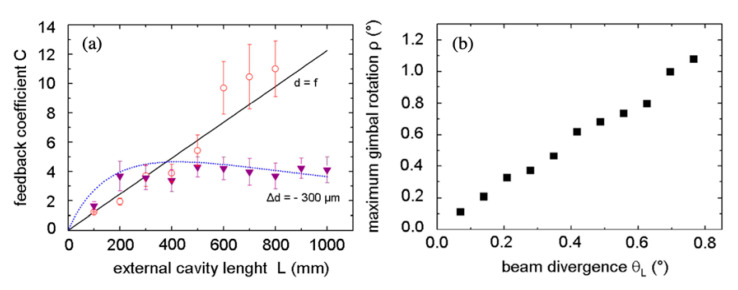
(**a**) Variation of feedback coefficient C for a collimated (d = f) and a divergent (Δd = −0.3μm) Gaussian beam. (**b**) Maximum measurable tilt angle of a target at 1 m distance from the laser emitting a divergent Gaussian beam (adapted from Reference [35]).

Up to six OF displacement sensors were closely packed in a 6 × 6 cm^2^ plate in order to simultaneously measure five misalignment degrees of freedom (two linear in the transverse plane and three angular around Cartesian axis) of a longitudinally sliding target along a 1 m long rail [33]. In order to optimize the feedback coupling across the long linear displacement without resorting to real-time adaptive optics in closed loop control [34], a passive and robust solution was adopted by shaping the mode-matching factor *ε* adjusting the actual divergence of the laser beams. The variation of the feedback coefficient C with target distance for a collimated and a slightly divergent Gaussian beam is compared in Figure 5. The moderate feedback regime could be maintained along the full slider range by setting out of focus the laser collimation optics. The corresponding divergent beams also tolerated the tilt of the target across the misalignment range of about 1 degree, as reported in Figure 5b. 

**Figure 6:**
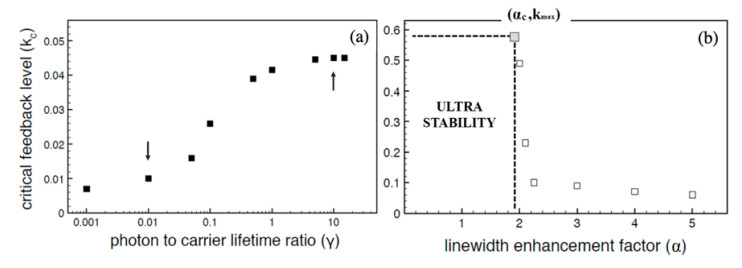
Dependence of the lowest feedback level *κ*_c_ necessary for triggering mode instability on the laser stationary emission on the lifetime ratio (**a**) and the linewidth enhancement factor (LEF) (**b**). Downward arrow in (**a**) marks typical value of semiconductor diode lasers, whereas upward arrow marks values of quantum-cascade lasers. (**b**) for γ = 10, the stationary solution remains stable irrespective of the feedback level at LEF values smaller than about 2 (adapted from Reference [28]).

In spite of the demonstrated robustness of the OF signal in SDLs, class B lasers are intrinsically more vulnerable to develop instabilities in case the feedback power crossed certain thresholds [36], due to undamped relaxation oscillations. On the other hand, class A lasers, such as quantum cascade lasers, are intrinsically more stable to OF level changes [28,37] providing a robust technological platform for developing OF sensors and systems. Two main factors contribute QCLs their stability against changes of the OF level: the small linewidth enhancement factor and the short carrier lifetime, both related to the unipolar nature of band transitions in QCLs. Figure 6 shows the calculated minimum feedback (*κ*_c_) level required to destabilize the stationary laser emission modes. The larger the carrier lifetime (Figure 6a), with respect to the photon lifetime, and the larger the LEF (Figure 6b), the smaller the critical feedback level *κ*_c_.

Among the many interesting features making QCLs appealing for OF sensing and imaging, are their narrower linewidth [38] and much larger voltage drop at the laser terminals, with respect to SDLs, making it easier to achieve a large SNR without resorting to external detectors.

### 3.2. Optical Feedback Sensing in Quantum Cascade Lasers

The following steps along the chosen path gain pace from the invaluable opportunity of OF interferometry of performing even better in the spectral regions where external detectors are less attractive because of their higher cost and poorer sensitivity. Commercially available QCLs cover almost continuously the full spectral range 3–11 μm, and state-of-art THz QCLs extend the range above 100 μm. (External) detector-less OF systems can be designed at pretty much any wavelength where a semiconductor laser exists with the added benefits of better SNR and disturbance-free the longer the emission wavelength is. More sophisticated OF sensing schemes are then adaptable to the mid infrared spectral range, acknowledging increased resolution, and sensitivity to chemicals absorption. 

**Figure 7:**
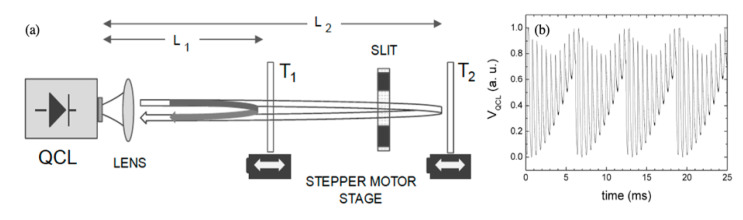
(**a**) Schematic setup illustrating the self-referenced OF principle. T_1_ is a semitransparent reference target sets in motion whose partial reflection modulates the OF signal from target T_2_, as shown in (**b**). The slit between T_1_ and T_2_ sets the relative feedback strength *κ*_1_/*κ*_2_ (adapted from Reference [39]).

A single-beam self-referenced OF scheme based on MIR-QCL, capable of differential speed measurement is demonstrated in Reference [39]. Figure 7 illustrates the basic working principle: a semitransparent reference target is inserted along the optical path and set to motion in order to adding a reference modulation superposed to the OF signal. The careful choice of the reference target parameters allowed for *λ*/100 resolution of the target displacement [29]. Once accounted for the longer wavelength of MIR-QCLs, this result compares favorably with the best resolutions achieved by OF in visible diode lasers, with the advantage of a much larger and stable SNR (Figure 7b). 

**Figure 8:**
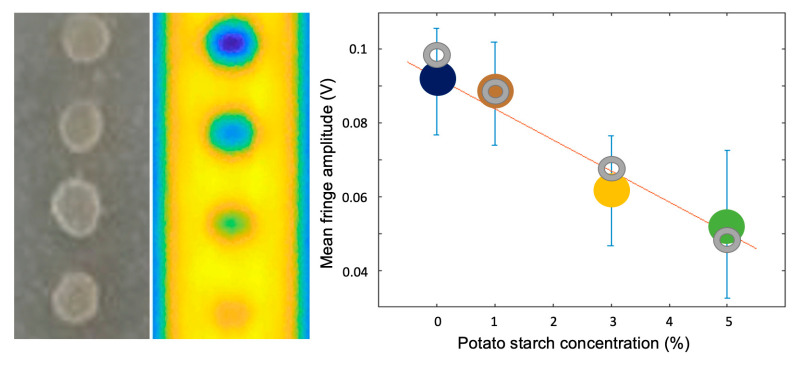
From left to right: photography and full-system simulated image of potato starch pills dispersed in PDMS matrix. Rightmost: measured (color bullets) and calculated (grey rings) fringe amplitude versus starch concentration averaged across the pills surface (adapted from Reference [41]).

The longer wavelengths made available by QCLs offer the opportunity to extend OF sensing to materials analysis [40] and chemical imaging, since most of the molecular distinctive absorption features occur in the mid infrared spectral range. One of the most relevant absorption band in biological materials, often addressed as a target to distinguish healthy from diseased tissues, is the amide-I band around 1550–1580 cm^−1^ (6.3–6.5 μm). Common potato starch also shows a broad absorption feature in the same range, due to carbon–oxygen bonds, and was used as a phantom to calibrate OF sensitivity to absorption change, in contrast to refractive index changes typically addressed by interferometric measurements. The calibration curve is reported in Figure 8, showing a relatively broad measurement range, with sensitivity limited by the signal amplitude fluctuations both due to inhomogeneous potato starch concentration and diffuse reflectance of the powdered samples. 

In closing this section, we recognize the footprints along the way from a multimodal sensing technology towards a versatile scanless and detectorless imaging system: (i) robust signals can be acquired at any available laser wavelength; (ii) cross-talk of adjacent OF channels is not relevant, especially when the signal is recorded as the laser terminals voltage; (iii) absorption as well as index changes can be detected; (iv) multiple parameters can be extracted from the OF signal; and (v) target inhomogeneity could partially spoil the OF sensitivity and requires special care.

## 4. Single Mode Feedback Imaging

The amplitude of the OF signal retains its dependence on the target optical properties (see Section 2) and scanning the laser spot across the sample surface enables imaging of the target refractive index distribution, of the extinction coefficient distribution, and ultimately of the target complex reflectivity. 

Alike all interferometric measurement, OF is primarily sensitive to phase changes along the whole light path from laser to target and return. In the previous section, the phase changes making up the detected OF fringes derived from the target displacement: Δ*ϕ* = 2*k*Δ*L*. For a stationary target Δ*L* = 0 = Δ*ϕ*. However, the OF signal can be recovered by superposing a reference modulation as shown in Figure 7 or, more conveniently, by direct modulation of light power, either by sweeping the laser driving current or by chopping the laser beam. Whereas both methods are applied in semiconductor lasers OF sensing, the latter is often preferred in QCLs system because of their stability against changes of the OF level (see Section 3.1) and their relatively large tuning coefficient Δ*λ*/Δ*I*. 

This second experimental section aims at illustrating the versatility of OF interferometry as imaging technology spanning the whole optical spectrum from 0.4 to 120 μm. Since we rely on phase-locking techniques, we make one step backward along axis 1, while moving upward one step along axis 3 in Figure 1, in going from single channel to multi-channel images.

### 4.1. Index Contrast

**Figure 9:**
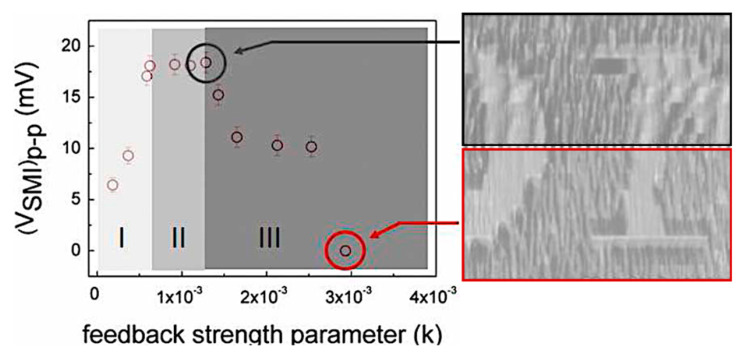
Left panel: fringe amplitude dependence on feedback level, expressed in term of the *κ*-parameter. Right panel: at low feedback level (upper half) OF fringes are clearly distinguishable superposed to the index contrast image, due to surface misalignment from the optic axis. At large feedback level (lower half) the OF signal loses phase information of the reflected beam and is defined by the feedback power only (adapted from Reference [43]).

Refractive index is by far the most relevant contrast mechanism in reflective coherent imaging modalities. Minute changes of the local refractive index modulate the phase front of the reflected beam enabling the tracing of the edges of the sample morphology. OF imaging often allows for two different regimes: a weak feedback regime where the fringe amplitude is determined by the optical phase and a strong feedback regime where the signal amplitude is determined by the optical power. An exemplary image recorded in those regimes is reported in Figure 9, where the central part of the word SONY printed onto a CD is recorded by a QCL at 6.2 μm. In case of a non-uniformly flat sample, additional OF fringes occur because of the effective Δ*L* ≠ 0 while scanning the surface. This additional information may actually be evaluated to add depth resolution to the OF images [42].

### 4.2. Absorption Contrast

**Figure 10:**
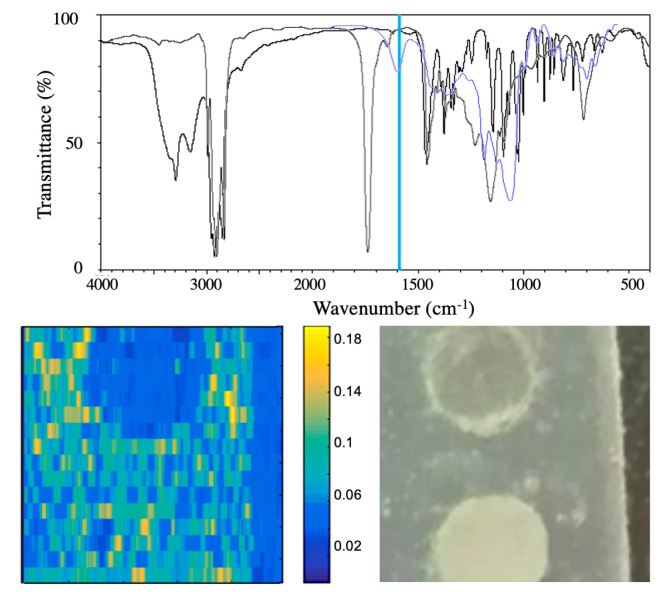
**Top**: transmittance infrared spectra of agarose (black), Intralipid^®^ (grey) and potato starch (blue). **Bottom**: images of potato starch (**upper**) and Intralipid^®^ (lower) pills in agarose matrix. Left panel shows the OF image taken at 6.2 μm by raster scanning the MIR-QCL beam. Right panel shows a photo of the sample (adapted from [41]).

The optical power contrast capability permitted in the strong feedback regime appears to be potentially valuable to expand the OF imaging by including the mapping of chemical elements dispersed in the sample. We already demonstrated in Section 3.2 the OF dependence of a resonant laser on the concentration of some absorbing chemical element. Figure 10 extends the spot measurement of Section 3.2 to scanning OF modality by showing the power contrast image due to selective absorption. Two pills, one of potato starch (absorbing at 6.2 μm) and one of Intralipid^®^ (transparent at 6.2 μm) in agar–agar matrix, were prepared and included into a dehydrated agar–agar matrix (transparent at 6.2 μm); OF signal of a MIR-QCL at 6.2 μm only detected the potato starch pill. The sample were prepared to be inhomogeneous and diffusive in order to mimic the scattering properties of real tissues. Back scattered light from this type of samples propagates with large speckle fluctuations lowering the image contrast and limiting the actual resolution. 

**Figure 11:**
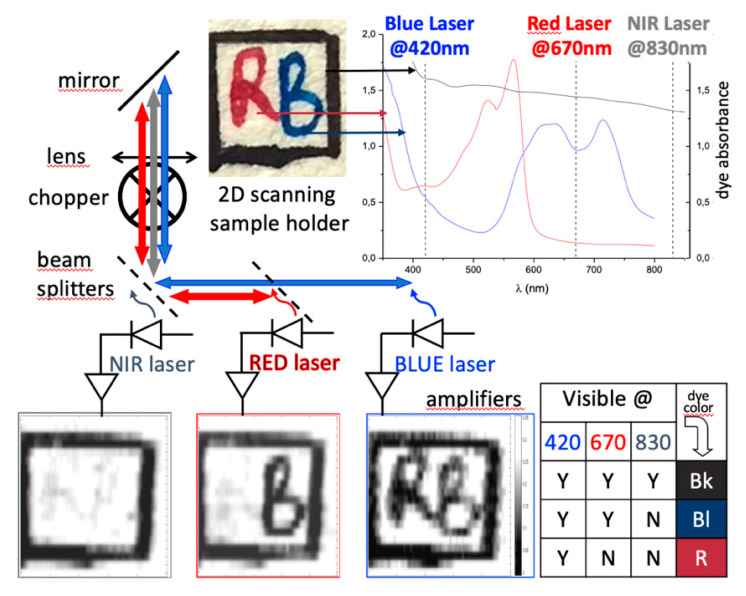
Graphic abstract of the experimental outcome. The lookup table at bottom-right helps to identify the color distribution on the target. (adapted from Reference [41])

A plain demonstration of chemicals selective imaging is shown in Figure 11. Three different visible SDLs were arranged to scan a simple target made of a watercolor paper printed with three pigmented inks having different absorption spectra. The proper choice of laser wavelengths and feedback levels make easy the unambiguous identification of the pigment under observation by direct comparison of the OF images. A systematic calibration of the system would allow interpolation and image fusion for quantitative analysis, although only on a relative scale.

### 4.3. Mixed Contrast

At the cost of a more intensive signal analysis, OF images provide a wealth of information about the complex dielectric susceptibility of the sample, enabling the mapping of materials properties at resolution comparable with more sophisticated technologies [44]. 

**Figure 12:**
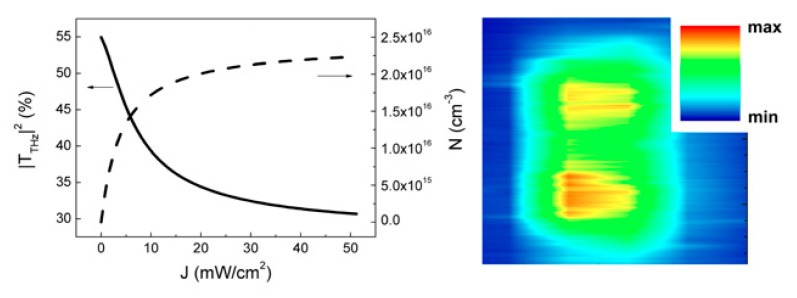
THz-QCL OF imaging of photogenerated carrier density distribution in n-doped Si (right, actual image is 1.6 × 1.6 mm^2^). Left panel shows calculated THz transparency at the carrier density excited by a cw laser diode (*λ*exc = 832 nm) (adapted from [45]).

High density locally photogenerated carriers in highly doped semiconductors modify the dielectric response of the material over the whole spectral range. We arranged a pump-probe experiment in n-doped Si wafers, pumping electrons in the conduction band by photoexcitation above the Si bandgap and probing the surface complex reflectance at THz wavelengths by OF imaging. Figure 12 shows the extracted stationary carrier density distribution in the Si top layer following photoexcitation by a TE_10_ shaped laser mode.

At variance with previous examples, where the OF signal was directly adapted to provide images, a theoretical model of the radiation-matter interaction including the laser/detector dynamics is required to mapping the desired information. Once available such a model, OF interferometry can be pushed very far, also competing with sub-diffraction resolution optical microscopy [14]. 

Several other examples can be found in literature of the different imaging modalities possible with OF schemes [46,47,48]. A recent review can be found in [49].

In closing this section, we appreciate the other steps taken along the way towards a versatile scanless and detectorless OF imaging system: (vi) direct images can be acquired in scanning mode at any available laser wavelength; (vii) voltage signals are far more insensitive to cross-talk than photocurrent signals admitting multispectral OF imaging; (viii) index contrast as well absorption contrast images can be acquired by setting different feedback levels; (ix) pump-probe experiments give OF access to full characterization of materials’ properties; (x) diffusive targets produce speckle patterns that changes randomly during beam scanning, lowering the image contrast and limiting the actual resolution.

## 5. Multimode Feedback towards Scanless Imaging

Although we have come quite a long way, we have been marching up to this point relying on one leg only, a semiconductor laser operating on single mode, as common practice in OF systems and modeling. It does not need to be that way. When more than one laser mode is available to the system, OF may trigger multi-mode instability in semiconductor diode lasers and reduce sensitivity, simulations involve heavier codes and larger computational resources, but multi-mode systems also open new scenarios for multi parametric sensing and scanless imaging modalities. The central idea behind this conception is that, in addition to the laser and target parameters defining the feedback level, an additional degree of freedom comes from shaping the mode-matching factor, which has been conventionally assumed constant but can be tailored on purpose, as already described in Section 3.1 for a single mode SDL.

The content of this last section is more suggestive than descriptive. There are very few examples in literature of OF in multi-mode lasers [50,51,52]. With the goal of scanless and detectorless multimodal imaging, we first explore opportunities foreseen in multi-transverse mode lasers (stepping one step further along axis 3 in Figure 1), and then conclude with a proof-of-principle that opens OF research to future developments, eventually reaching the block (1.2, 2.3, 3.3), which is at hands with the farthest block from our starting point.

VCSELs are intrinsically multi-transverse mode, even relatively small area VCSELs show polarization dynamics, often accompanied by spatial mode switching. In the simplest case of only two spatial modes we showed that spatially shaped feedback can be tailored to simultaneously detect two orthogonal degrees of freedom of motion with a single OF module [26,53]: longitudinal displacement (on-axis) and transverse rotation (roll). A larger number of transverse modes can be accommodated in broad area VCSELs, whose complex dynamics can be regulated by incorporating a phase coupling mechanism inside of the cavity structure or by providing a suitable external feedback. Frequency detuned feedback, like that at work in OF Doppler velocimetry, is one way to change the laser mode configuration, as demonstrated in Reference [54]. Photonic-crystal VCSELs, as they are called, might then work as phase-sensitive CCDs taking scanless OF phase-contrast images as conventional CCDs take single-shot intensity-contrast images. Initially available only at near-infrared wavelengths, phase-coupled QCL arrays were recently realized also in the THz spectral range [55].

However, 2D detectors, either in form of coupled transverse modes in broad area lasers or independent pixel matrix [56,57,58], are not required to take scanless OF imaging. New computational techniques aimed to the extrapolation of images from low-resolution detectors have reached commercial maturity [59]. Compressive sensing (CS) algorithms allow to reconstruct an N-pixel image taking less than N intensity measurements. CS can be pushed up to the realization of single pixel cameras, which produce spatially resolved image employing a bucket detector. The laser cavity of a single mode semiconductor laser is an inherently single-pixel detector and a scanless OF image can be produced via CS techniques.

**Figure 13:**
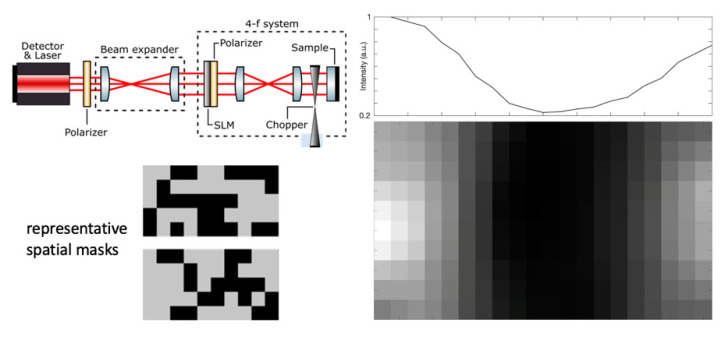
OF scanless image (bottom right) of a metal wire on a mirror surface, taken with the setup sketched at top-left. The OF laser source and detector is an SDL (*λ* = 670 nm). The spatial-light modulator is taken from a video projector. Total imaged area 2 × 4 mm^2^. Two representative masks adopted for illuminating the target are shown at bottom-left. The integral over pixel columns of the wire image is shown at top-right (adapted from [60]).

We recently demonstrated a proof-of-principle of this approach, as shown in Figure 13. The beam traveling in the OF interferometer is expanded to the size of the target surface and its intensity profile is controlled by a spatial light modulator (SLM). The CS algorithm evaluates the feedback for different intensity profiles and extrapolates an image of the object. 

Extension of the CS imaging technology to the mid-infrared and THz wavelengths is far more interesting because of lack of sensitive and affordable 2D detectors in those spectral regions and the stronger stability of QCLs. 

The spatial resolution of scanless images is partly related to the pixel size of the spatial modulator and partly to the number of implemented masks. Both liquid crystals and micro-mirrors SLMs have relatively large pixels and slow reconfiguration time, thus enforcing a resolution-speed compromise on image quality. Both these limitations could be partly relieved by using optically reconfigurable metasurfaces, especially effective at long wavelength, as we demonstrated in Reference [61] by modifying the complex reflectivity of a Si wafer by photoexcitation of free carriers with subwavelength periodicity. 

## 6. Conclusions

The retrospective narrative of our research in the field of OF has come to a point where all players have been characterized and we are ready to script the next season. Without spoiling the coming episodes, we can anticipate two directions of emerging attentiveness: integrated Si-photonics and in vivo biomedical imaging. 

On the one hand, the robustness and flexibility of OF sensors adaptable for kinematic measurements of proximity, deformation, distance, displacement, and velocity, make them interesting for applications in robotics [62]. Innovative OF sensors based on quantum-dot lasers, which are intrinsically more stable than SDLs [63], can be integrated in “all-in-one” Silicon Photonics (SiPh) chips allowing for cascaded or parallel multi-parametric sensing schemes on a single technology.

On the other hand, our understanding of cancer in all tissues indicates that the structure as well as biochemical, mechanical, and functional behavior is altered. No single feature is a diagnostic indicator, but their correlates combined, should enhance the diagnostic power of an examination. Multimodality imaging is the next frontier in early diagnosis and screening, especially in skin and epithelial tissues cancers, which have among the lowest survival rates, if not recognized at their earliest stage. OF imaging has the potential of optically addressing a number of physical parameters characterizing malignant lesions with respect to surrounding tissue and benign lesion [4,21,64]. 

## 7. Patents

EP2479533A1 Scamarcio, G.; Mezzapesa, F.P.; Ancona, A.; Sibillano, T.; De Lucia, F.; Dabbicco, M.; Lugarà, P.M. Laser system for ablation monitoring; US8234081 Dabbicco, M.; Intermite, A.; Ottonelli, S.; Radisavljevic, B.; Scamarcio, G. System for optical fiber strain measurement; WO2010000283 Dabbicco, M.; De Lucia, F.; di Vietro, M.; Florio, C.; Mezzapesa, F.P.; Ottonelli, S.; Plantamura, M.C.; Polito, S.; Scamarcio G. System for laser measurement of the target motion.
